# The Role of HMGB1 in Rheumatic Diseases

**DOI:** 10.3389/fimmu.2022.815257

**Published:** 2022-02-17

**Authors:** Yuanji Dong, Bingxia Ming, Lingli Dong

**Affiliations:** Department of Rheumatology and Immunology, Tongji Hospital, Tongji Medical College, Huazhong University of Science and Technology, Wuhan, China

**Keywords:** HMGB1, alarmin, rheumatic diseases, autoimmunity, damage-associated molecular pattern

## Abstract

HMGB1, a highly conserved non-histone nuclear protein, is widely expressed in mammalian cells. HMGB1 in the nucleus binds to the deoxyribonucleic acid (DNA) to regulate the structure of chromosomes and maintain the transcription, replication, DNA repair, and nucleosome assembly. HMGB1 is actively or passively released into the extracellular region during cells activation or necrosis. Extracellular HMGB1 as an alarmin can initiate immune response alone or combined with other substances such as nucleic acid to participate in multiple biological processes. It has been reported that HMGB1 is involved in various inflammatory responses and autoimmunity. This review article summarizes the physiological function of HMGB1, the post-translational modification of HMGB1, its interaction with different receptors, and its recent advances in rheumatic diseases and strategies for targeted therapy.

## Introduction

High mobility group proteins were first extracted and identified from the bovine thymus in 1973 (1). Then it was further divided into HMGA, HMGB, and HMGN3 families (2), and among them, the HMGB family included high mobility group box 1 (HMGB1), HMGB2, and HMGB3. HMGB1 is highly conserved in evolution, with 99% homology between rodents and human amino acid sequences (3). In the 1990s, the DNA binding domain in the amino acid sequence of HMGB1 was found to play an essential role in maintaining the structure of chromatin and regulating gene transcription (4, 5). In some cases, HMGB1 can be transferred from the nucleus to the cytoplasm and extracellular to perform immunological functions (6). Extracellular HMGB1 as a danger-associated molecular pattern, alone or with partners, activates multiple receptors such as the receptor of advanced glycation end-products (RAGE), toll-like receptor 2 (TLR2), and TLR4 to participate in proliferation, tissue repair, inflammation, and cell death (7). Furthermore, HMGB1 is closely related to sterile inflammation and can promote autoimmune diseases as an endogenous adjuvant (8). Because of its potential povital function, increasing research has been paid to the role of HMGB1 in inflammation and autoimmune diseases. Various strategies targeting HMGB1 have also been developed, including neutralizing antibodies, truncated HMGB1 box A protein, soluble RAGE (sRAGE), and small molecule inhibitors. This review mainly describes the new advances of HMGB1 in rheumatic diseases.

## Biological Function of HMGB1

### Structural Characteristics of HMGB1

**Figure 1:**
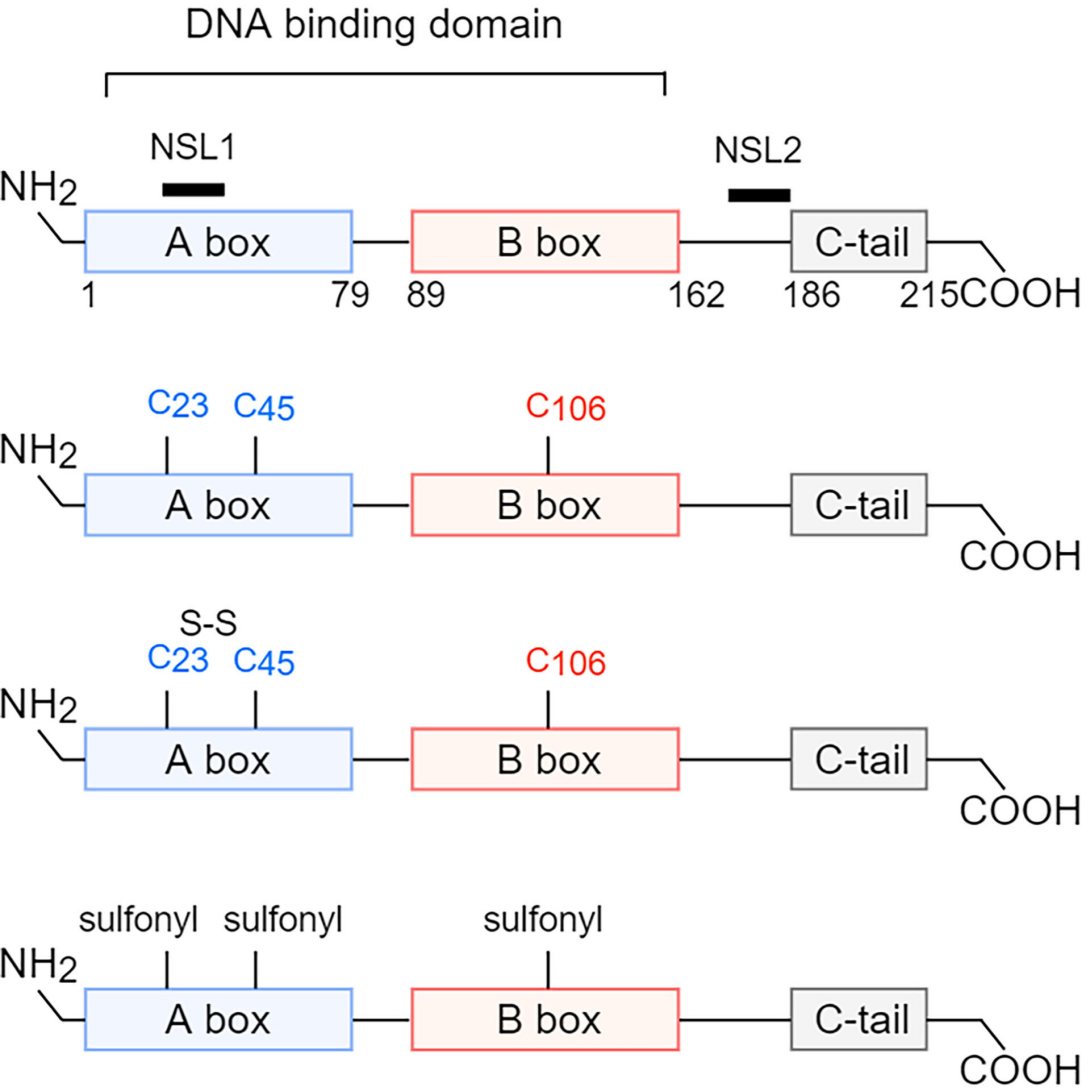
Structure and redox reaction of HMGB1. HMGB1 is composed of A box, B box, c-terminal tail, two nuclear localization sequences (NLS1, 27-43 AA; NLS2, 178-186 AA), and two homologous L-type DNA-binding regions. HMGB1 has three cysteine residues, Cys23, Cys45, and Cys106, and has three different redox forms. When all three cysteine residues are in reduced form, the main extracellular function of HMGB1 is chemotaxis. When Cys23 and Cys45 form intramolecular disulfide bonds, and Cys106 is in reduced form, the main extracellular function of HMGB1 is to promote the production of proinflammatory factors. When all three cysteine residues are oxidized, the main extracellular function of HMGB1 is unable to induce inflammation. NLS, nuclear localization sequences; AA, amino acid. Cys, cysteine.

HMGB1 is highly conserved in evolution, and the only difference between human and murine in the amino acid sequence of HMGB1 is that human Glu189 and Asp202 is replaced by Asp189 and Glu202 in mice, respectively (7). HMGB1 contains 215 amino acid residues, including two nuclear localization sequences (NLS1, 27-43AA; NLS2, 178-186AA), two homologous L-type DNA-binding regions (A-box and B-box), and one acidic C-terminal tail (containing aspartic acid and glutamic acid repeats) **(** **Figure 1** **)**. The B box of HMGB1 showed a pro-inflammatory effect, while the A box displayed an anti-inflammatory effect by the antagonism of the B box (9, 10). The A box and B box of HMGB1 are positively charged with three helices, they are both capable of nonspecific binding to DNA. In addition, HMGB1 has a DNA secondary structure-specific binding site being an essential structure for binding deformed DNA, which can loosely bind to the small slot of DNA in a non-sequence-dependent manner (11). The structural characteristics of HMGB1 enable it to play a role in the nucleus and allow it to be released into the cytoplasm and extracellular to perform more complex functions.

### Different Modifications and Redox State of HMGB1

HMGB1 can be modified post-translationally by different enzymes. Typical modifications include acetylation, phosphorylation, methylation, adenosine diphosphate (ADP), ribosylation, and N-glycosylation (11). The first three modifications affect the binding ability of HMGB1 with DNA, and modification by poly (ADP-Ribosyl) results in the inhibition of efferocytosis of macrophages (12). In activated monocytes, the acetylation of lysine residues in NLS resulted in the translocation of HMGB1 from the nucleus to the cytoplasm (13, 14). Classical protein kinase C-mediated phosphorylation of NLS was also crucial for cytoplasmic localization of HMGB1 (15). In addition, methylation of HMGB1 leading to the cytoplasmic translocation of HMGB1 was observed in neutrophils (16). The cytoplasmic HMGB1, lacking a leader sequence, could only be secreted into the extracellular region by the non-classical lysosomal pathway (6).

In addition, there are three cysteine residues (Cys23, Cys45, and Cys106) in the amino acid sequence of HMGB1, and the redox status of HMGB1 depends on the form of the three cysteine residues in different tissue microenvironments (17). Cys23 and Cys45 can form disulfide bonds, while Cys106 can only be in a reduced state or be oxidized alone. So, there are three forms of isomers **(** **Figure 1** **)**. When the three cysteine residues were in the thiol state (reduced type), HMGB1, by interacting with CXCL12, could induce leukocyte chemotaxis *via* CXCR4 (7, 18). When Cys23 and Cys45 formed disulfide bonds and Cys106 was in reduced form, HMGB1 could interact with TLR4 to exert a pro-inflammatory effect (19). Mutation without forming disulfide bonds or further oxidation of the disulfide isoform could abolish the ability of HMGB1 to induce cytokine production (20). When Cys23, Cys45, and Cys106 were all oxidized, the function of sulfonated HMGB1 could not induce inflammation (7). This is consistent with the situation that Cys106 is in the reduced state during necrosis and in the oxidation state during apoptosis. Furthermore, homo-dimerization of HMGB1 at Cys106 has been found in the nucleus and extracellular, but its biological significance remains unclear (21).

### Release Characteristics of HMGB1

**Figure 2:**
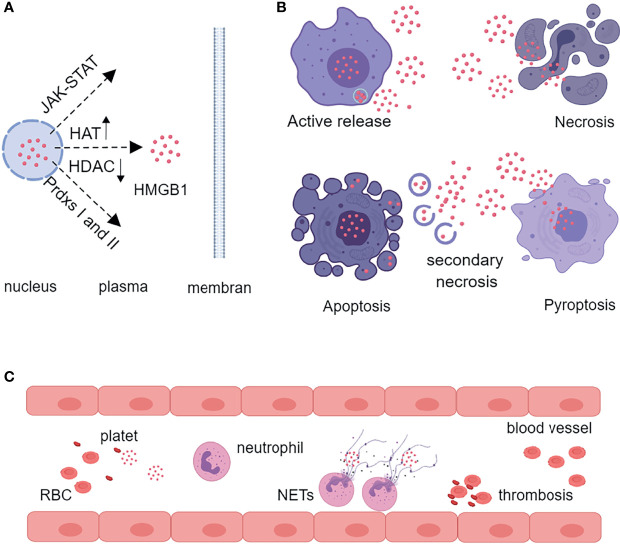
Characteristics of HMGB1 release. **(A)** HMGB1 is translocated from the nucleus to the cytoplasm by the JAK-STAT pathway, by increasing the activity of HAT and decreasing the activity of HDAC, or by oxidation of nuclear peroxiredoxins I or II. **(B)** Activated inflammatory cells can actively secrete HMGB1 through the lysosomal pathway. Necrosis, pyroptosis, and secondary necrosis following apoptosis can also release HMGB1. **(C)** Activated platelets produce large amounts of HMGB1 to promote thrombosis and neutrophil release of NETs. HAT, histone acetylase; HDAC, histone deacetylase; Prdxs, peroxiredoxins; RBC, red blood cell; NETs, neutrophil extracellular traps.

HMGB1 is located in the nucleus and can be released during cell activation or death. Activated immune cells [macrophages, dendritic cells (DCs)] and tissue cells (endothelial cells neurons, astrocytes) actively secreted HMGB1 (21). The release of HMGB1 from the nucleus to the cytoplasm depends on the activation of the Janus kinase (JAK) signal transducer and activator of transcription 1 (STAT1) pathway, or the balance of the histone acetylase (HAT) activity and histone deacetylase (HDAC) activity, or the formation of disulfide HMGB1 *via* peroxidase I and II (14, 21). Then HMGB1 was packaged in vesicles and released extracellular *via* lysosomal pathway (6). In addition, different forms of cell death could cause the release of HMGB1. Necrotic cells could passively release large amounts of disulfide HMGB1. Pyroptosis, characterized by the activation of inflammasome and caspase-1/caspase-11, could produce reduced and disulfide isomers of HMGB1 (22, 23). Under normal circumstances, apoptotic cells did not release HMGB1. When phagocytes failed to clear the apoptotic cells, secondary necrosis occurred, which resulted in the release of sulfonated and disulfide HMGB1, so this process was also harmful to the host (24, 25). In addition, activated platelets produced large amounts of disulfide isomers of HMGB1 to promote thrombosis and neutrophil activation (26–28) **(** **Figure 2** **)**.

### Interaction With Different Receptors and Cleavage of HMGB1

A total of 15 types of HMGB1 receptors were described in the literature (29). RAGE was the first HMGB1 receptor to be discovered. HMGB1 with other pro-inflammatory partner molecules could interact with RAGE to enter the endosomal and lysosomal system, then HMGB1 disrupted the lysosomal membrane at low pH, and the partner molecules bound to homologous receptors in the cytosol to mediate the synthesis of pro-inflammatory mediators (21). HMGB1 could also interact with TLRs (TLR2, TLR4) to activate the NF-κB and IRF pathways and then produce cytokines and chemokines for the inflammation and immune response (30). When HMGB1 bound to the TIM-3 on DCs, it blocked the anti-tumor effects of DNA vaccines and chemotherapy drugs (30). HMGB1 also synergistically stimulated N-methyl-D-aspartate receptor (NMDAR) receptors with IL-1β, leading to calcium influx in the central nervous system or peripheral tissues (31). The HMGB1-CXCR12 complex could also bind to CXCR4 to recruit inflammatory cells to damaged tissues (18). HMGB1 promoted the interaction between RAGE on endothelial cells and Mac-1 on neutrophils and then neutrophil recruitment (32). In addition to its pro-inflammatory effects, HMGB1 mediated anti-inflammatory effects under certain circumstances. HMGB1 interaction with CD24 and siglec-10 (siglec-G in mice) inhibited the activation of NF-κB and prevented cytokine release (33). Disulfide HMGB1 inhibited the release of inflammatory cytokines in sepsis by binding to haptoglobin to induce heme oxygenase-1 (HO-1) and IL-10 production in a CD163^+^ dependent manner (34). HMGB1 interacts with various receptors, playing different roles in immunity and inflammation.

There are also enzymatic cleavage sites in the structure of HMGB1, such as that cathepsin G cleaved HMGB1 rapidly (within 5 min) *in vitro*, suggesting rapid extracellular clearance of HMGB1 under inflammatory conditions (35). HMGB1 was also predicted to be degraded by other proteases, but further validation was needed. Studies about HMGB1 cleavage are meaningful to provide new therapeutic strategies for various diseases.

### Immunological Characteristics of Extracellular HMGB1

**Figure 3:**
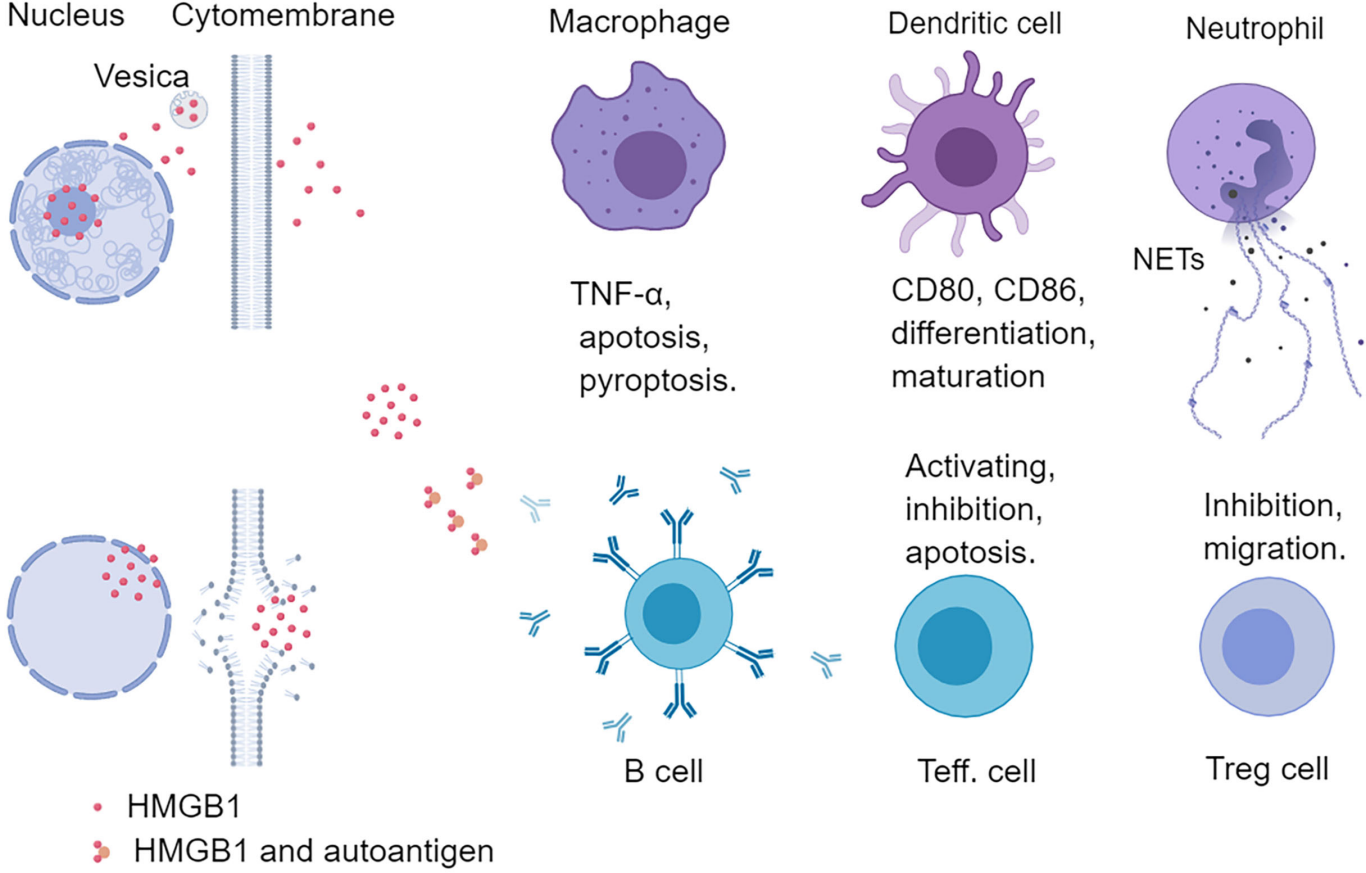
Effects of HMGB1 on immune cells. HMGB1 can regulate both innate and adaptive immunity. HMGB1 can promote the release of pro-inflammatory factors from macrophages and induce apoptosis and pyroptosis of macrophages. HMGB1 can promote the differentiation and maturation of DCs and up-regulate the level of costimulatory molecules (CD80, CD86) of DCs. HMGB1 also promotes the neutrophil release of NETs, and HMGB1 is also abundant in NETs. HMGB1 can form a complex with a nucleic acid to promote the recognition of innate immune cells and T helper cells and stimulate B cells to secrete autoantibodies. Different concentrations of HMGB1 can exert different functions on effector T cells. Low concentration HMGB1 promotes the activation of T cells, while high concentration HMGB1 inhibits and even induces T cell apoptosis. HMGB1 can also directly act on Treg cells to promote their migration and survival. Teff., effector T cell; NETs, neutrophil extracellular traps.

The extracellular HMGB1 not only mediated the repair of muscle tissue (skeletal muscle and cardiac muscle tissue) but also regulated many kinds of innate immune cells (neutrophils, macrophages, DCs) and adaptive immune cells (effector and regulatory T cells) **(** **Figure 3** **)** (36). HMGB1 promoted neutrophil migration and amplified neutrophil activity to accelerate the formation of neutrophil external traps (NETs) to aggravate tissue damage (37, 38). Lipopolysaccharide (LPS) stimulated macrophages released HMGB1 and HMGB1 alone or combined with LPS further activated macrophages (39, 40). HMGB1 not only activated macrophages to produce chemokines and inflammatory factors but also induced macrophage apoptosis in a dose- and time-dependent manner (41, 42). In addition, HMGB1 was involved in the pyroptosis of macrophages (43) and the maturation and differentiation of DCs (44–46). HMGB1 enhanced the sensitivity of mature DCs in response to CCL21 and then the migration to lymph nodes, and HMGB1 secreted by mature DCs up-regulated the costimulatory molecules level (CD80, CD83, and CD86) in an autocrine manner (8, 47). In addition to the effect on innate immune cells, HMGB1 could also directly act on T lymphocytes. HMGB1 had a dual impact on T lymphocytes, increasing CD4^+^ T lymphocyte, especially CD4^+^Th17 activity at low concentration, while inhibiting T lymphocyte activity at high concentration (36, 48, 49). In addition, HMGB1 was related to T cells apoptosis and mitochondrial apoptosis (50). Although HMGB1 was beneficial to the migration and survival of regulatory T cells, it inhibited the activity of regulatory T cells by RAGE or TLR4 pathway (51, 52). Further study showed that HMGB1 significantly down-regulated the expression of Foxp3 and cytotoxic T lymphocyte-associated antigen-4 (CTLA-4) on regulatory T cells of the spleen in mice (53). These results indicate that HMGB1 has an extensive effect on immune cells and is involved in the disease process of inflammatory response.

## HMGB1 in Rheumatic Diseases

Rheumatic diseases are characterized by the enhanced autoimmune response and the production of autoantibodies. Although the research on the pathogenesis of rheumatic diseases continues to deepen and the biological agents continue to break through, new therapeutic targets still need to be explored. In the past 20 years, the role of HMGB1 in rheumatic diseases has been extensively studied. Here we summarize the characteristics and new insights of HMGB1 in rheumatic diseases **(** **Table 1** **)**.

**Table 1:** The role of HMGB1 in rheumatic diseases.

Disease	The role of HMGB1 in disease pathogenesis	References
RA	HMGB1 levels were increased in the serum, synovium, and synovia. HMGB1 levels in serum of RA patients were higher than that of OA patients and correlated with disease activity scores. HMGB1 promoted osteoclast and proinflammatory factor production and accelerated the activity of metalloproteinases and plasminogen activators. HMGB1 synergized with CXCL12 in active RA patients contributing to the influx of pro-inflammatory cells. In the model of CIA, HMGB1 was also involved in neurogenic inflammation.	(54–62)
SLE	HMGB1 levels in serum of SLE patients were increased and correlated with disease activity scores. High levels of HMGB1 converted monocytes into M1 type, promoted inflammation, and reduced the clearance of apoptotic cells. HMGB1 could also activate pDC and mDC and promote the release of NETs from neutrophils. HMGB1 could promote the rapid and abundant aggregation of ALD-DNA in macrophages through clathrin/alveolar protein-1. Serum HMGB1 could be used as a biomarker of NPSLE.	(63–69)
IIM	HMGB1 levels in serum of PM and DM patients were higher than that of healthy controls and higher in patients with Interstitial lung disease. Patients with high levels of HMGB1 had lower overall survival and disease-free survival. HMGB1/RAGE axis was involved in the amyloid deposition in muscle tissue of IBM patients. HMGB 1 could promote inflammation muscle fatigue and induce expression of MHC1 molecules in the early stage of the disease but promote the protection and regeneration of muscle tissue in the late stage of the disease.	(70–76)
SS	The expression of HMGB1 was increased in labial glandular tissue and serum of SS patients. Serum HMGB1 levels were higher in SSA positive or extra-glandular involvement. Treatment with anti-HMGB1 antibodies improved xerostomia and xerophthalmia in mouse models.	(77–81)
SSc	HMGB1 and sRAGE levels were elevated in SSc patients and mouse models. Platelets-derived particles expressed HMGB1, which promoted autophagy of neutrophils, enhanced proteolytic enzyme activity, and generated neutrophils extracellular traps. HMGB1 promotes the expression of α2AP in fibroblasts and contributes to tissue fibrosis. HMGB1 may be an independent risk factor for SSC-ILD or a new biomarker for SSc patients.	(82–87)
AS	HMGB1 levels in peripheral blood of AS patients were increased. HMGB1 levels were associated with disease activity scores, inflammatory markers, and HMGB1 receptor expression in PBMC. HMGB1 could be used as a laboratory indicator to reflect the therapeutic response of AS. Extracellular HMGB1 stimulated the expression of RANK in macrophages and promoted the differentiation of osteoclasts.	(88–91)
AAv	Serum HMGB1 levels were higher in AAV than healthy controls. HMGB1 enhanced the ability of neutrophils to burst, degranulate, and form NETs. HMGB1 increased the level of meosin in the GEnC and promoted the injury of GEnC. HMGB1 enhanced the proliferation of B cells and TLR9 levels in plasma cells in PBMC from patients with AAV, and the latter was positively correlated with Birmingham vasculitis activity score.	(92–99)
LVV	One study found that HMGB1 levels did not change in healthy controls and LVV patients, as well as during disease activity and remission, while another found HMGB1 levels increased in patients with Takayasu arteritis.	(100, 101)
MVV	HMGB1 levels in children with KD were higher. HMGB1 levels in patients with PAN were higher and positively correlated with hypersensitivity-CRP, serum creatinine, and 24-hour proteinuria.	(101, 102)
BD	HMGB1 levels in peripheral blood of BD patients were significantly increased.	(103, 104)
AOSD	Serum HMGB1 levels in AOSD patients were higher than those in healthy controls and correlated with CRP levels and the systemic score. Levels of serum HMGB1 were also found to decrease after the patient’s disease activity subsided. HMGB1 presented in the NETs of patients with skin lesions and high fever of AOSD patients.	(105, 106)
Gout	The expression of HMGB1 in PBMC of patients with acute gout was elevated. Macrophages stimulated by MSU resulted in the translocation and release of HMGB1. The absence of C5aR2 inhibits the activation of NLRP3 inflammasome and the release of HMGB1.	(107, 108)

RA, rheumatoid arthritis; SLE, systemic lupus erythematosus; IIM, idiopathic inflammatory myopathy; SS, Sjögren’s Syndrome; SSc, systemic sclerosis, AS, ankylosing spondylitis; AAV, Anti-neutrophil cytoplasmic antibody (ANCA)-associated vasculitides; LVV, large vessel vasculitis; MVV, medium vessel vasculitis; BD, Behcet’s disease; AOSD, Adult-onset still disease. OA, osteoarthritis; CIA, collagen-induced arthritis; ALD-DNA, activated lymphocyte-derived DNA; NPSLE, neuropsychiatric systemic lupus erythematosus; RAGE, the receptor of advanced glycation end-products; ILD, interstitial lung disease; RANK, receptor activator of nuclear factor-κB; NETs, neutrophil external traps; GEnC, glomerular endothelial cell; KD, Kawasaki disease; PAN, polyarteritis nodosa.

### Rheumatoid Arthritis

Rheumatoid arthritis (RA) is a kind of erosive arthritis involving the synovium. It is a common autoimmune disorder and is often accompanied by extra-articular symptoms (109). Immune disorders of RA include the production of autoantibodies, tissue infiltration of effector T cells, impaired function of the tissue-protective macrophages, and the transition of synovial stromal cells into pathogenic cells (54). However, current treatments still only slow the progression of the disease. There are several studies about the relationship between HMGB1 and RA. It has been reported that HMGB1 levels were increased in synovial tissue and synovial fluid of RA patients, and HMGB1 concentration in serum of RA patients was higher than that of osteoarthritis (OA) patients and was related to disease activity score (55–59). Furthermore, Cecchinato et al. found that vascular and synovial cells produced high levels of Thioredoxin-1 and Thioredoxin reductase, accompanied with local COX2/PEG2 and JAK/STAT signaling cascades to promote the activity of the CXCL12/HMGB1 heterocomplex on monocyte maintaining the inflammatory condition (60). In the mouse model of collagen-induced arthritis (CIA), immunohistochemical staining of synovial tissues revealed HMGB1 expression in various cell types, including fibroblast, synovial cell, macrophage and vascular endothelial cell, with significant cytoplasmic and extracellular localization (110). HMGB1 induced the expression of hypoxia-inducible factor 1α (HIF-1α) and vascular endothelial growth factor (VEGF) in synoviocytes of RA patients *in vitro* while neutralizing antibody treatment decreased the level of HIF-1α and angiogenesis (111). Furthermore, HMGB1 participated in osteoclast formation and pro-inflammatory factors production and accelerated the activity of metalloproteinases and tissue fibrinogen activators (62, 110). In addition, HMGB1 was involved in neurogenic inflammation. In the CIA model, HMGB1 released by nociceptors (peripheral sensory neurons) exacerbated inflammation and pain responses in peripheral tissues (61). All these results suggest that HMGB1 plays a pivotal role in RA and is a potential target for the therapy of RA.

### Systemic Lupus Erythematosus

Systemic lupus erythematosus (SLE) is a chronic autoimmune disorder with multiple organ involvement and unknown etiology. SLE affects the patient’s skin, joint, heart, lung, gastrointestinal system, and nervous system, leading to tissue damage and clinical symptoms (63). Serum HMGB1 levels in SLE patients were elevated and correlated with disease activity (112). In addition, anti-HMGB1 autoantibodies could be detected in the serum of SLE patients, and anti-A-box antibodies showed high specificity for SLE. These two autoantibodies were related to the Systemic Lupus Erythematosus Disease Activity Index (SLEDAI) and anti-double strand DNA (anti-dsDNA) antibody level (64). High levels of HMGB1 induced macrophage polarization towards the M1 phenotype, which reduced the clearance of apoptotic cells (65). HMGB1 also promoted the activation of pDC and mDC, which up-regulated pro-inflammatory factors (IL-1β, IL-6, and TNF-α) and costimulatory molecules (HLA-DR, CD40, and CD86) production (66, 67). Furthermore, HMGB1 from the NETs of neutrophils was positively associated with the progression of lupus nephritis (113). HMGB1 could combine with other molecules (nucleic acid, IgG, immune complex, etc.), which stimulated innate immunity to produce inflammatory factors and type 1 interferon to exacerbate autoimmune response (114). In the skin lesions of SLE patients, HMGB1 expression was increased and positively correlated with TNF and IL-1β level, and UV radiation increased levels of cytoplasmic and extracellular HMGB1 in the skin (115, 116). In patients with lupus nephritis (LN), HMGB1 induced proliferation of glomerular mesangial cells through TLR2, and HMGB1^+^ microparticles of urine could distinguish active and inactive LN (117, 118). *In vitro* and *in vivo* studies showed that HMGB1 increased the production of inflammatory cytokines in renal macrophages through RAGE (68, 119). In addition, HMGB1 promoted the rapid and abundant accumulation of lymphocyte-derived DNA (ALD-DNA) *via* clathrin/caveolin-1, and activated ALD-DNA promoted macrophage activation in LN (69). Serum HMGB1 levels were also increased in patients with psychiatric lupus and were positively correlated with disease activity but had little effect on psychotic lupus-related seizures. Serum HMGB1 could be used as a biomarker in neuropsychiatric systemic lupus erythematosus (NPSLE). In addition, in patients with psychiatric lupus, impaired blood-brain barrier led to the entry of anti-DNA antibodies into the central nervous system (CNS). The anti-DNA antibodies subset could cross-react with NMDAR to impair spatial memory. Further studies found that extracellular HMGB1 was also bound to NMDARs and formed a C1q-HMGB1-NMDARs complex on the dendrites of neurons. The complex interaction with RAGE/TLR4 on microglia led to neuronal dendrite damage and cognitive dysfunction (120). These results suggest that targeting HMGB1 in SLE is promising, but more investigations are needed.

### Idiopathic Inflammatory Myopathy

Idiopathic inflammatory myopathy (IIM) is a group of autoimmune diseases that affect the striated muscle, including polymyositis (PM), dermatomyositis (DM), and inclusion body myositis (IBM) (70). Serum HMGB1 levels were elevated in PM and DM patients and higher in IIM patients with interstitial lung disease. In addition, patients expressing high levels of HMGB1 had lower overall and disease-free survival rates (71, 72). The pro-inflammatory effect of HMGB1 in myositis was mainly mediated by TLR4. In the mouse model of experimental autoimmune myositis (EAM), TLR4 and HMGB1 were elevated in affected muscle tissue. Treatment of the PBMC of EAM mice with TLR4 or HMGB1 antibodies down-regulated the expression of TNF, IL-6, and MHC-I (73). Extracellular HMGB1 also promoted muscle fatigue through TLR4 in patients and mice with myositis (74). But HMGB1 promoted amyloid deposition by acting on RAGE in patients with IBM (75). In patients with myositis, HMGB1 presented with cytoplasmic and extracellular translocation in both endothelial cells and infiltrated immune cells. After corticosteroid treatment, the cytoplasmic and extracellular HMGB1 in inflammatory cells were decreased, while the staining intensity of endothelial cells was similar before and after treatment (76).

On the other hand, the injection of HMGB1 in muscle tissue promoted the regeneration of muscle and blood vessels (121). These results suggest that HMGB1 plays a dual role in idiopathic myositis. In the early stage, HMGB1 promoted inflammation by up-regulating the expression of MHC-I molecule and also accelerated muscle fatigue. In contrast, during the progressive phase, HMGB1 promoted the regeneration of muscle tissue. However, further experiments are still needed to confirm, especially the mechanism of HMGB1 in promoting muscle tissue regeneration.

### Sjögren’s Syndrome

Sjögren’s Syndrome (SS) is characterized by exocrine glands involvement, which presents with xerophthalmia and xerostomia. This disease can also show extra-glandular manifestations and even B cell lymphoma. The treatment for SS includes artificial tears, artificial saliva, glucocorticoid, and immunosuppressants (77). Ek et al. found that extracellular HMGB1, TNF-α, and IL-1β were increased around the infiltrated mononuclear cells by staining the labial glandular tissue in SS patients. TNF-α and IL-1β could promote the secretion of HMGB1 from monocytes, and HMGB1, in turn, acted with RAGE and TLR4 to further induce the release of TNF-α and IL-1β (78). Dupire et al. found that serum HMGB1 levels were increased significantly compared with healthy controls and were higher in SS patients with positive SSA autoantibody (79). In another study, serum HMGB1 and sRAGE were elevated and associated with the EULAR Sjögren’s Syndrome Disease Activity Index (ESSDAI) in patients with SS, and HMGB1 levels were much higher in patients with extra-glandular involvement (80). Studies in the mouse model found that subconjunctival injection of anti-HMGB1 antibody improved the symptoms of ocular dryness by regulating the level of innate lymphoid cells 3 in draining lymph nodes. Intraperitoneal injection of the anti-HMGB1 antibody also alleviated xerostomia by downregulating the HMGB1/TLR4/NF-kB signaling pathway and improved aquaporin protein 5 expression (81, 122). Although the detailed mechanism of SS needs to be further confirmed, therapies targeting HMGB1 are promising.

### Systemic Sclerosis

Systemic sclerosis (SSc) is a systemic autoimmune disease characterized by vasculitis and fibrosis, mainly affecting the skin and internal organs (82). Ayumi et al. found that serum levels of HMGB1 and sRAGE were increased in SSc patients and were higher in patients with organ involvement and immune abnormalities. In addition, serum HMGB1 levels were positively correlated with Modified Rodnan total skin thickness score and negatively correlated with lung function (83). In the bleomycin-induced mouse scleroderma model, the HMGB1 and sRAGE levels in peripheral blood were increased compared with control mice (83). Norma et al. further explored the source of HMGB1 in the vascular system of SSc patients. They observed that platelet-derived particles expressing HMGB1 might be involved in microvascular injury and continuous activation of endothelial cells (84). Subsequently, oxidized HMGB1 in the blood of SSc patients could promote the activation of neutrophils (85).

Furthermore, it has been reported that activated platelets from SSc patients could interact with neutrophils by promoting autophagy to up-regulate the activity of proteolytic enzymes and neutrophil extracellular traps (NETs) production. However, these changes on neutrophils were reduced after the treatment of HMGB1 A box (28). In another bleomycin-induced mouse scleroderma model, local M2-macrophage-derived HMGB1 contributed to the development of tissue fibrosis by producing α2-antiplasmin *via* RAGE receptors on fibroblasts (86). Zheng et al. found that serum calpain activity and HMGB1 levels were significantly higher in SSc patients with interstitial lung disease (ILD) than those in non-ILD SSc patients. Serum calpain activity and HMGB1 levels might be independent risk factors for SSc-ILD or novel biomarkers for patients with SSc (87). These results suggest that HMGB1 plays an essential role in the development and progression of SSc. In the future, the therapeutic efficacy of targeting HMGB1 in SSc patients should be reasonably evaluated to provide more evidence for clinical transformation.

### Ankylosing Spondylitis

Ankylosing spondylitis (AS) is a chronic inflammatory rheumatic disease that mainly affects the spine and sacroiliac joints (123). Severe cases could develop spinal deformity and rigidity. The initial study found that serum HMGB1 levels were increased in patients with AS but were not associated with erythrocyte sedimentation rate (ESR), C-reactive protein (CRP), Bath Ankylosing Spondylitis Disease Activity Index (BASDAI), Bath Ankylosing Spondylitis Functional Index (BASFI), or ASQoL scores (123). However, our previous work found that HMGB1 levels were not only increased in AS patients but also significantly positively correlated with BASDAI, Ankylosing Spondylitis Disease Activity Score (ASDAS), BASFI, CRP, and ESR, and were correlated with the expression of HMGB1 receptors such as TLR2, TLR4, and IL-1RACP in the PBMC of AS patients (89). These differences may be related to the sample size and gender composition. A follow-up study of 147 patients with AS who were treated with TNF-α inhibitor or oral non-steroidal anti-inflammatory drug plus sulfasalazine revealed a consistent trend in disease activity with the level of HMGB1 before and after treatment, which suggest that HMGB1 could reflect the disease activity of AS to some extent and could be used as a laboratory indicator to reflect the therapeutic response (90). Hou et al. further found that the ratio of oxidized low-density lipoprotein and low-density lipoprotein (ox-LDL/LDL) was increased in peripheral blood of AS patients, and ox-LDL induced cytoplasmic translocation of HMGB1. Extracellular HMGB1 induced receptor activator of nuclear factor-κB (RANK) expression in CD68 monocyte by reacting with RANKL to induce its differentiation into osteoclast (91). Although the study about the role of HMGB1 in AS is still limited, the existing research has proved that HMGB1 participated in the development of AS. Further research and clinical trials are needed to support these results.

### Systemic Vasculitides

Systemic vasculitides are a group of diseases characterized by inflammation and fibrinoid necrosis of vascular walls or perivascular tissue. Anti-neutrophil cytoplasmic antibody (ANCA)-associated vasculitis (AAV), Takayasu arteritis (TA), giant cell arteritis (GCA), polyarteritis nodosa (PAN), Kawasaki disease (KD), and Behcet’s disease (BD) all belong to the category of systemic vasculitis (124). The role of HMGB1 has been extensively studied in AAV, and the level of HMGB1 in the serum of AAV patients was higher than that in healthy controls (92, 93). Further studies found that serum HMGB1 levels were higher in patients with renal involvement or granulomatous presentation (93–96). Subsequent studies found that HMGB1 enhanced the ability of neutrophils to burst, degranulation, and form NETs after ANCA stimulation, and HMGB1 was positively correlated with endothelial activation marker (sICAM-1, VEGF) levels, as well as HMGB increased the level of meosin protein in the glomerular endothelial cell (GEnC), which participated in the cross-reaction of anti-MPO antibody and promoted the injury of GEnC (97, 98). In addition, HMGB1 enhanced the TLR9 levels and proliferation of B cells in plasma cells from PBMC of patients with AAV, and the latter was positively correlated with Birmingham vasculitis activity score (99). The research on HMGB1 in other vasculitides is few. De Souza et al. found that serum HMGB1 levels in TA and GCA patients were comparable to those in healthy controls, and there was no difference between active and remission stages (100). Another study found that peripheral blood HMGB1 level was higher in TA patients than in healthy controls (101). Hoshina et al. found that the HMGB1 levels in children with KD were higher than that in healthy controls, and the highest levels were in the acute phase with a gradual decrement after defervescence (102). Zhu et al. found that the serum HMGB1 levels in PAN patients were significantly higher than those in AAV and TA patients and positively correlated with hypersensitivity-CRP, serum creatinine, and 24-hour proteinuria (101). Ahn et al. found serum HMGB1 levels were elevated in BD patients and even higher in patients with intestinal involvement but were not correlated with disease activity (103). Another study found HMGB1 levels were not different among patients in the active disease, patients receiving treatment, and patients in remission without treatment (104). These results provide evidence for targeting HMGB1 in the treatment of vasculitis, but more relevant studies and clinical trials are needed to verify.

### Other Rheumatic Diseases

Adult-onset Still disease (AOSD) is a rare multigenic systemic autoinflammatory disorder characterized by high fever, rash, joint pain, hepatosplenomegaly, and elevated white blood cells in peripheral blood. It has been reported that serum HMGB1 level was higher in patients with AOSD than that in healthy controls, especially those with rashes and sore throats. This increased HMGB1 levels were related to CRP and the systemic score (105). However, serum HMGB1 levels were decreased in the follow-up patients with reduced disease activity (105). Another study found that higher HMGB1 levels were observed in peripheral blood NETs of AOSD patients with skin lesions and a high fever (106).

Gout is an inflammatory joint disease caused by the deposition of uric acid crystals in the joints. It has been reported that the transcription levels of IL-1β, IL-18, caspase-1, and HMGB1 in PBMC of patients with active gout were significantly higher than those in non-active patients or healthy controls. Monosodium urate crystal (MSU) stimulation of U937 macrophages resulted in the translocation and release of HMGB1. Inhibition of caspase-1 by siRNA could reduce the MSU-induced release of HMGB1 (107). Another study reported that a lack of C5AR2 inhibited the activation of the NLR family, pyrin domain-containing protein 3 (NLRP3) inflammasome, and the release of HMGB1 in a mouse model and that C5AR2 activated MAPK, ERK, and type 1 interferon pathways to amplify dsRNA-dependent protein kinase R expression to promote the activation of NLRP3 inflammasome (107). These results suggest that HMGB1 is involved in AOSD and gout in a pro-inflammatory fashion and that targeting HMGB1 may be a promising therapeutic approach.

## Targeting HMGB1 Therapy

Currently, various strategies that inhibited HMGB1 expression, release, and associated signaling have been investigated in the literature, including neutralizing HMGB1 antibodies, HMGB1 A box protein, peptide P5779, glycyrrhizin, resveratrol, platinating agent (like cisplatin), quercetin, dexmedetomidine, ethyl pyruvate, thrombomodulin, haptoglobin, metformin, diflunisal, sRAGE, triptolide, etc. (39, 125). Targeting HMGB1 therapy has been extensively studied in sepsis, ischemia-perfusion injury, organ transplantation, and tumors (126–129). In rheumatic diseases, anti-HMGB1 mAb (m2G7) was a relatively well-studied antibody that played an anti-inflammatory role in collagen-induced arthritis (130). This m2G7 could bind to the 53-63 amino acids of the HMGB1 sequence and impede the interaction of HMGB1 with RAGE or TLR4 to reduce inflammation (130). Intrathecal injection of m2G7 could relieve chronic pain in the model of collagen-induced arthritis (131). The treatment of m2G7 had no effect on lupus nephritis in MRL/LPR mice, but another monoclonal antibody to HMGB1 has been reported to play a beneficial role (132, 133). In addition, neutralizing HMGB1 antibodies have been reported to alleviate xerostomia and xerophthalmia in mouse models (81, 122). Recombinant Box A protein has been used *in vivo* to antagonize HMGB1 induced cell migration, leukocyte recruitment, and inflammation and has been shown to play a beneficial role in animal models of IBM and experimental arthritis (39, 75, 134). Future treatment needs to be considered both in blocking the functional epitopes of HMGB1 and inhibiting the binding ability of HMGB1 to other molecules to eliminate the role of HMGB1 as a pro-inflammatory and endogenous adjuvant.

## Discussion

HMGB1 is a multi-functional protein and plays a pivotal role in regulating transcription, inflammation, and repair. HMGB1 plays critical biological functions alone and forms a complex with exogenous substances such as LPS to enhance immune response and form a complex with endogenous substances such as nucleic acid or autoantigen to act as an endogenous adjuvant to mediate the inflammatory and autoimmune response. Thus, targeting HMGB1 is a promising therapeutic strategy for treating rheumatic diseases. However, we should also recognize the complexity of the role of HMGB1 in rheumatic diseases and the difficulty of extracellular HMGB1 detection. Only by breaking through these limitations and detecting the level of HMGB1 more sensitively can we ensure that targeting HMGB1 will bring more benefits to patients with rheumatic diseases.

## Author Contributions

YD wrote the article and drew the figures. YD, BM, and LD organized and revised the paper. All authors reviewed and approved the submitted version.

## Funding

This work was supported by grants from the National Natural Scientific Foundation of China (No. 81771754 and No. 81901586) and Tongji Hospital Clinical Research Flagship Program (No. 2019CR206).

## Conflict of Interest

The authors declare that the research was conducted in the absence of any commercial or financial relationships that could be construed as a potential conflict of interest.

## Publisher’s Note

All claims expressed in this article are solely those of the authors and do not necessarily represent those of their affiliated organizations, or those of the publisher, the editors and the reviewers. Any product that may be evaluated in this article, or claim that may be made by its manufacturer, is not guaranteed or endorsed by the publisher.
